# Translation, adaptation, and validation of ASK nasal-12 into Brazilian Portuguese

**DOI:** 10.1016/j.bjorl.2024.101511

**Published:** 2024-09-14

**Authors:** Jeniffer Cristina Kozechen Rickli, Ricardo Landini Lutaif Dolci, Leandro Meira Martins, Miguel Bosenbecker Böhm, Dino Rafael Perez, Américo Rubens Leite dos Santos, Paulo Roberto Lazarini

**Affiliations:** a Santa Casa de São Paulo, Departamento de Otorrinolaringologia, São Paulo, SP, Brazil; b Faculdade de Ciências Médicas da Santa Casa de São Paulo, São Paulo, SP, Brazil

**Keywords:** SNOT-22, Questionnaire, Skull base, ASK12, Quality of life

## Abstract

**Objective:**

Translate, adapt, and validate the Anterior Skull Base Nasal Inventory 12 questionnaire (ASK Nasal-12) to Brazilian Portuguese.

**Methods:**

A prospective longitudinal observational study was developed at the Skull Base Surgery Center clinic of the Irmandade Santa Casa de Misericórdia de São Paulo. There were five stages to translating the original ASK-12 questionnaire: two independent translations, consensus between the two, back-translation, comparison, and revision for the semi-final version. Internal consistency, reliability, and validation with a previous already validate questionnaire were all assessed.

**Results:**

Over 30 months 72 patients in total were included in the study, of which six had to be excluded afterwards according to the research criteria. The internal consistency using Cronbach’s Alpha was calculated and successful. Next the reliability and agreement between the test and retest were analyzed. The Minimally Important Clinical Difference (MCID) was calculated for the 13 scores at each of the four time points. Finally, the final translated version was validated with the SNOT-22 questionnaire.

**Conclusion:**

The translation, adaptation, and validation of the ASK Nasal-12 questionnaire into Brazilian Portuguese appear consistent and effective regarding its cultural equivalence for monitoring the QOL of patients undergoing transnasal access for endoscopic anterior skull base surgeries.

**Level of evidence:**

II.

## Highlights

• A prospective longitudinal observational study.
• Translate, adapt, and validate ASK Nasal-12 to Brazilian Portuguese.
• The final translated version was validated with the SNOT-22 questionnaire.
• Specific questionnaire to anterior skull base endonasal surgery.
• Assessment of QOL after skull base endonasal surgery is essential.

## Introduction

Quality of Life (QOL) is considered a multidimensional construct encompassing an individual’s perception of their well-being at a specific moment. Its measurement must cover physical, functional, psychological, and social aspects.^1^ The assessment of QOL is not the same as the assessment of the symptom, given that the same symptom can have significantly different impacts on two people or two occasions on the same individual.^2^, ^3^, ^4^

Skull-based surgeries have made significant progress in recent decades, and refinements in the technique and surgical approach have improved survival rates and transformed the assessment of QOL in these patients. This made it a more relevant factor,^2^ with a search for better rates by the surgeon, in addition to the goal of tumor removal.

Traditionally, research has focused on surgical outcomes, such as the extent of resection, mortality rate, complications, and cure rate, all crucial markers of clinical outcome. However, they do not statistically explain factors inherent to QOL, such as pain control, social well-being, cognitive symptoms, emotional health, and physical and aesthetic appearance.^1^, ^5^

Identify specific needs and ensure that surgeons are always looking for a better and less invasive surgical technique and, when possible, an improvement in the patient’s QOL.^2^, ^4^

Improving or preserving QOL in surgeries for patients with skull base injuries presents several challenges, such as proximity to several vital structures, which can lead to high morbidity and worsened QOL. Skull base tumors are amenable to a variety of surgical approaches, such as endoscopic approaches, open or mixed, each of which with different implications on QOL.^6^

Currently, transnasal endoscopic surgery, depending on the tumor location, is the choice to approach these conditions, and one of the main morbidities is the manipulation of the nasal region.^7^ The nasal approach deserves extreme attention, as patients with disease in the skull base generally have normal functioning of the nasal mucosa before the procedure and, after treatment, they present complaints such as nasal obstruction, rhinorrhea, and changes in smell which, although they may be transient, affect QOL.^5^, ^8^

The promising development of the transnasal endoscopic technique expanded the need for an instrument to measure the nasal complaints inherent to the procedure. In 2012, Little et al. presented the Anterior Skull Base Nasal Inventory 9 questionnaire – (ASK nasal-9), with nine questions relating to the perioperative quality of life in endoscopic anterior skull base surgeries scaled as a basis, the frequency of symptoms.^9^ In 2013, by the same author, the first nasal morbidity assessment instrument specific to endonasal skull base surgery was published, the Anterior Skull Base Nasal Inventory 12 questionnaire (ASK Nasal-12), to complement other multidimensional QOL scales.^10^

The Nasal ASK-12 is a self-reported, one-dimensional, and specific assessment of sinonasal symptoms, such as pain, nasal obstruction, changes in smell, and scabs’ formation after endonasal surgery. It is measured using a six-point Likert system where a higher score on any of the questions indicates a worse QOL for the patient. This questionnaire proved to be sensitive to clinical changes and was validated according to psychometric parameters.^4^, ^10^

Since the ASK Nasal-12 is a questionnaire developed in English, it is necessary to be translated into Brazilian Portuguese for it to be used properly. However, a simple literal translation of the questionnaire may not be satisfactory due to cultural and language differences between people. Considering this fact, the scenario is set to translate and adapt the nasal ASK-12 for Brazilian practice.^11^, ^12^

The aim of this study is to translate, adapt, and validate the QOL questionnaire for anterior skull base surgery – nasal ASK-12 – from English to Brazilian Portuguese.

## Methods

A prospective longitudinal observational study was developed at the Skull Base Surgery Center clinic of the Irmandade Santa Casa de Misericórdia de São Paulo (NCBC - ISCMSP) in the care of patients undergoing surgical planning and postoperative follow-up, authorized by the Ethics Committee and Research of the institution (CAAE 04812918.7.0000.5479).

### Translation

In the first phase, the ASK Nasal-12 quality of life questionnaire was translated into 5 main stages.^13^, ^14^, ^15^

**Figure 1:**
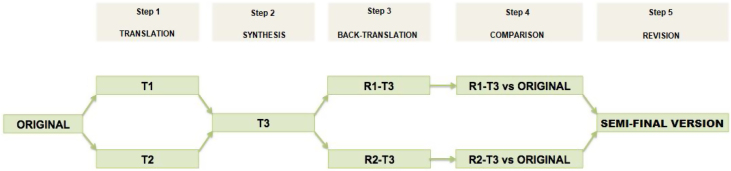
The ASK Nasal-12 quality of life questionnaire was translated into 5 main stages.

1 Translation into Brazilian Portuguese was done openly and independently by two otorhinolaryngologists, subspecialists in rhinology, with proficiency in the English language, generating two versions (T1 and T2).
2 Consensus between the two translations and another bilingual translator co-author of the research to prepare a single version (T3).
3 Back-translation of version T3 done independently by two native English speakers proficient in Brazilian Portuguese, generating two-second versions in the original language (R1‒T3 and R2‒T3).
4 Review and compare the back-translations with the original questionnaire by a translation and re-translation committee, with an evaluation of the content, semantics, and concept and preparation of the pre-final translation by the committee made up of translators, re-translators, and co-authors.
5 Review and analysis of conceptual, semantic, and content equivalence to prepare the pre-final version (Fig. 1).

### Cultural adaptation

Individuals over 18-years-old monitored at the clinic NCBC - ISCMSP were selected. Patients who agreed to participate in the research signed the Informed Consent Form (ICF) before participating.

The standardization of the questionnaire application was recommended for all participants. After satisfactory test application, the participants also responded regarding their understanding of the items asked, writing down the doubts raised.

Given the difficulties in reading and understanding the text among the population assisted in this service, we chose to read the questionnaire to the participants to avoid possible biases with the self-administration of the original questionnaire.

A new meeting with the translation and back-translation committee was necessary to adapt the changes suggested during the process. Thus, the final version of this questionnaire was created, which was applied to other patients also monitored at this clinic.

### Statistical analysis

The final version of the questionnaire was applied to patients attending the ISCMSP Skull Base clinic in 4 stages: preoperatively, 1-month, 3-months, and 6-months postoperatively.

The statistical analysis process was done in three stages:

1) Internal consistency: We used Cronbach’s Alpha technique to analyze the internal consistency of the protocol questions.
2) Reliability: We analyze at least 20% of the sample by applying a retest, and thus, using the ICC (Intraclass Correlation Coefficient), we evaluate the reliability and/or agreement between the test and the retest. We also calculated the MCID (Minimum Clinically Important Difference).
3) Validation: We carried out validation with an already existing score (validation of the construct). Spearman Correlation was used to validate the ASK-12 using the value of the SNOT-22,^12^ a questionnaire already translated and validated into Brazilian Portuguese.

### Inclusion criteria

- Patients who are monitored at NCBC - ISCMSP clinic.
- Patients scheduled for transnasal endoscopic skull base surgery.
- Older than 18-years-old.

### Exclusion criteria

- Patients who have already undergone a previous surgical procedure using the transnasal endoscopic.
- Patients who have already undergone a previous (open) craniotomy for the same reason as a transnasal endoscopic indication.
- Patients who undergo transnasal endoscopic surgery, and require a new surgical approach, through the same way or via craniotomy (open).
- Patients who do not want to participate in the research.
- Patients who have undergone radiotherapy.

## Results

Over 30 months, 62 patients were followed up in this study, of which 6 patients had to be excluded according to the research criteria, and 10 patients with malignant sinonasal and skull base tumors were included to complete the ASK-12 – questionnaire and SNOT-22.

### Final version of the translation and adaptation of the ASK-Nasal 12 questionnaire

After the translation, synthesis, back-translation, comparison, and review phases regarding semantic equivalence, conceptual, and idiomatic, we reached the pre-final version of the questionnaire, which was applied to a pilot group of patients, which comprised 32.14% of the sample, in test and retest mode and accompanied by an assessment scale of understanding by the individual. Cultural adaptation is necessary when a sample greater than 15% of interviewees consider the question difficult to understand. Among the 12 questions presented in the pre-final version of the ASK Nasal-12 questionnaire, only question number 5 was considered difficult to understand by the sample; 21% of participants did not understand the sentence, making cultural adaptation necessary for this item. Furthermore, no difficulties or cultural barriers were detected in this pilot group, thus confirming the final version of the questionnaire.

### Anterior skull base nasal questionnaire – ASK Nasal-12

**Figure 2:**
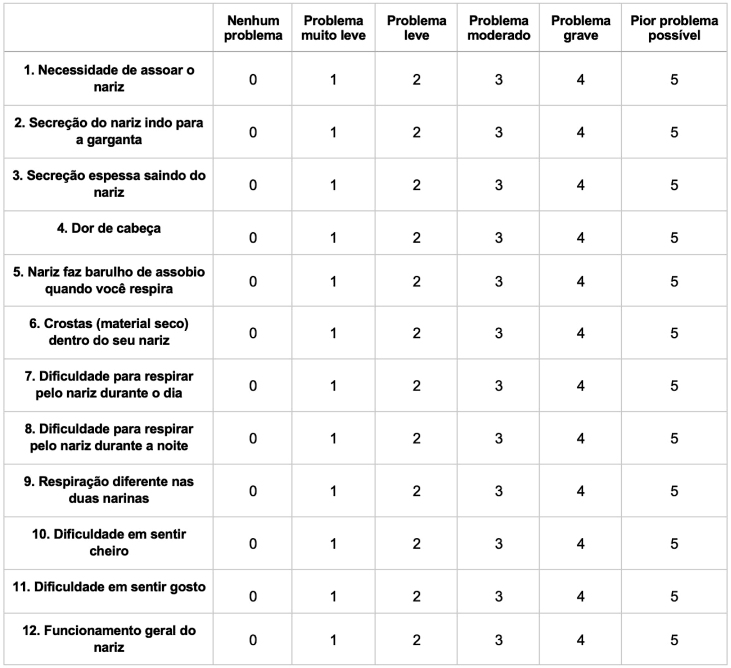
Final version of the translation and adaptation of the ASK-Nasal 12 questionnaire.

Below, you will find a list of questions about your symptoms. We will give you this questionnaire before and after surgery so that we can provide you with better treatment and monitor your progress. When making a choice, evaluate the frequency and severity of these symptoms. Please assess these problems according to what you have experienced over the past two weeks (Fig. 2).

### Statistical results

The first analysis was to define the internal consistency of the questionnaire using Cronbach’s Alpha at each of the four collection moments (Table 1).

**Table 1:** Internal Consistency of the ASK-12 Questionnaire (Cronbach’s Alpha).

	**Alfa**
**Pré**	0.926
**T1**	0.876
**T3**	0.838
**T6**	0.766

Next, the reliability and agreement between the test and retest were analyzed, which was applied in 18 cases, representing 32% of the sample. Thus, the ICC was established for each of the domains and in the total score (Table 2).

**Table 2:** Test-retest reliability for the ASK-12 questionnaire in 18 patients.

	**ICC**	**p-valor**
**Necessidade de assoar o nariz**	1.000	<0.001
**Secreção do nariz indo para a garganta**	0.997	<0.001
**Secreção espessa saindo do nariz**	0.949	<0.001
**Dor de cabeça**	0.994	<0.001
**Nariz faz barulho de assobio quando você respira**	1.000	<0.001
**Crostas (material seco) dentro do seu nariz**	0.622	0.031
**Dificuldade para respirar pelo nariz durante o dia**	1.000	<0.001
**Dificuldade para respirar pelo nariz durante a noite**	0.993	<0.001
**Respiração diferente nas duas narinas**	1.000	<0.001
**Dificuldade de sentir cheiro**	1.000	<0.001
**Dificuldade de sentir gosto**	1.000	<0.001
**Funcionamento geral do nariz**	1.000	<0.001
**ASK-12 Total**	0.996	<0.001

ICC, Intraclass Correlation index.

The Minimally Important Clinical Difference (MCID) was calculated for the 13 scores at each of the four time points. This value represents the minimum value that must be obtained in evaluating the outcome of a treatment to be considered a benefit to the patient (Table 3).

**Table 3:** Minimum clinically important difference from the ASK-12 Questionnaire.

		**Média**	**Desvio Padrão**	**N**	**SEM**	**DMCI**
**Necessidade de assoar o nariz**	Pré	0.70	1.58	56	0.21	0.59
	T1	2.11	1.14	56	0.15	0.42
	T3	1.13	0.95	56	0.13	0.35
	T6	0.38	0.52	56	0.07	0.19
**Secreção do nariz indo para a garganta**	Pré	0.80	1.66	56	0.22	0.61
	T1	2.07	1.16	56	0.15	0.43
	T3	1.16	1.09	56	0.15	0.40
	T6	0.45	0.57	56	0.08	0.21
**Secreção espessa saindo do nariz**	Pré	0.25	0.81	56	0.11	0.30
	T1	2.11	1.11	56	0.15	0.41
	T3	1.14	1.07	56	0.14	0.40
	T6	0.20	0.55	56	0.07	0.20
**Dor de cabeça**	Pré	1.32	1.62	56	0.22	0.60
	T1	1.71	1.44	56	0.19	0.53
	T3	0.98	1.17	56	0.16	0.43
	T6	0.38	0.62	56	0.08	0.23
**Nariz faz barulho de assobio quando você respira**	Pré	0.18	0.58	56	0.08	0.21
	T1	0.66	1.00	56	0.13	0.37
	T3	0.14	0.44	56	0.06	0.16
	T6	0.05	0.30	56	0.04	0.11
**Crostas (material seco) dentro do seu nariz**	Pré	0.11	0.31	56	0.04	0.12
	T1	3.02	1.14	56	0.15	0.42
	T3	1.61	1.04	56	0.14	0.38
	T6	0.54	0.66	56	0.09	0.24
**Dificuldade para respirar pelo nariz durante o dia**	Pré	0.46	1.08	56	0.14	0.40
	T1	1.00	1.21	56	0.16	0.45
	T3	0.30	0.74	56	0.10	0.27
	T6	0.05	0.23	56	0.03	0.08
**Dificuldade para respirar pelo nariz durante a noite**	Pré	0.77	1.39	56	0.19	0.51
	T1	2.21	1.41	56	0.19	0.52
	T3	0.91	1.15	56	0.15	0.43
	T6	0.21	0.41	56	0.06	0.15
**Respiração diferente nas duas narinas**	Pré	0.54	1.13	56	0.15	0.42
	T1	0.93	1.13	56	0.15	0.42
	T3	0.38	0.62	56	0.08	0.23
	T6	0.04	0.19	56	0.03	0.07
**Dificuldade de sentir cheiro**	Pré	0.50	1.21	56	0.16	0.45
	T1	2.57	1.48	56	0.20	0.55
	T3	1.30	1.56	56	0.21	0.58
	T6	0.57	1.32	56	0.18	0.49
**Dificuldade de sentir gosto**	Pré	0.27	0.88	56	0.12	0.33
	T1	1.34	1.55	56	0.21	0.58
	T3	0.59	1.04	56	0.14	0.39
	T6	0.13	0.38	56	0.05	0.14
**Funcionamento geral do nariz**	Pré	0.86	1.58	56	0.21	0.58
	T1	2.55	1.08	56	0.14	0.40
	T3	1.32	1.05	56	0.14	0.39
	T6	0.38	0.62	56	0.08	0.23
**ASK-12 Total**	Pré	6.38	10.32	56	1.38	3.82
	T1	21.73	9.15	56	1.22	3.39
	T3	10.63	7.30	56	0.98	2.70
	T6	3.82	4.11	56	0.55	1.52

DMCI, Minimum Clinically Important Difference; N, Number of patients included in the study.

The last step of the process was to validate the ASK-12 questionnaire with another already existing questionnaire and validate it for follow-up. The SNOT-22 questionnaire was considered an excellent comparative assessment for nasal symptoms. Spearman’s correlation to validate the ASK-12 using the SNOT-22 value was applied. Of the 56 patients included in the study, the SNOT-22 value was performed in 18, and 10 patients with malignant tumors of the nasal cavity or skull base were added, totaling 28 patients. (In all cases, the SNOT-22 questionnaire was applied at four moments to have a larger and more reliable sample, counting 112 moments).

The correlation (where it is written “Corr”, but which can also be denoted by *ρ* or *r*) is a value that varies from −1 to 1. When the correlation is positive, it means that as one variable increases its value, the other correlated to this also increases proportionally. However, if the correlation is negative, the variables are inversely proportional; as one increases, the other decreases, or vice versa.

It is concluded that there is a statistically significant correlation in the preoperative period where the *r*-value = 0.460 (*p*-value = 0.016). We also found a significant correlation in the 6^th^ month with *r* = 0.496 (*p*-value = 0.085), in addition to the General (4 periods), where the result of *r* = 0.364 (*p*-value < 0.001), and in the General without the Pre (1^st^, 3^rd^ and 6^th^ month) with *r* = 0.341 (Table 4).

**Table 4:** Correlation between ASK-12 and SNOT-22 for validation.

	**Corr (r)**	***p-*valor**
**Pre**	0.460	0.016
**1º month**	-0.112	0.584
**3º month**	0.131	0.524
**6º month**	0.496	0.085
**General (4 times)**	0.364	<0.001
**General (1º, 3º e 6º month)**	0.341	0.005
**General (1º e 3º month)**	0.172	0.224

## Discussion

Endonasal surgeries to access the anterior base of the skull temporarily modify the nose’s functionality, which, in many cases, is not a complaint or problem for the patient preoperatively. The possibility of having an instrument that checks and monitors this process is of great value in optimizing postoperative follow-up and being more assertive regarding the QOL obtained for these patients.

The ASK Nasal-12^9^ questionnaire is an excellent analytical tool for monitoring the QOL of patients undergoing transnasal surgeries for the anterior skull base; however, it is still available in English and has not yet been translated, adapted, and validated into Brazilian Portuguese.

In the pre-and postoperative follow-up of 56 patients, it was noticed that this series of twelve questions helped identify and act more assertively on nasal complaints related to the proposed surgical access, thus optimizing the QOL of the patients in this sample.

At all times of the collection, Cronbach’s Alpha was classified as excellent; that is, the protocol is, in fact, statistically consistent. It was noted that pre-surgery had the highest result, with an Alpha of 0.926, which is consistent with a sample without many preoperative nasal symptoms.

In all ASK Nasal-12 domains, a significant ICC was observed with very high values, which shows that this instrument has good reliability and agreement.

The MCID values for each score and at each time point were studied. In ASK-12 Total, there is an MCID of 3.82 in the preoperative period, which gradually decreases throughout the postoperative collections until it reaches 1.52 in 6-months, consistent with nasal health during the anterior skull base surgery via transnasal.

In this sample, there was a statistically significant validation of the ASK-12 by SNOT-22 when comparing the 4 moments together (preoperative and postoperative at 1, 3, and 6 months). The SNOT-22 questionnaire was used as a comparison as it is already validated and adapted to Brazilian Portuguese and used to assess the quality of the specific nasal site. This questionnaire was validated to evaluate functional endoscopic surgeries but is widely used in studies to analyze QOL in endonasal skull base surgeries. Therefore, the correlation in the present study to validate a specific questionnaire for endoscopic skull base surgery is highly reliable.^7^, ^8^

The correlation between the SNOT-22 and the ASK-12 was evaluated in the preoperative period, 6-months postoperatively, and in a general analysis that included the preoperative period and 1, 3, and 6-months after surgery. However, when analyzing the first and third months isolated, no statistically significant correlation was found. In the authors' opinion, this lack of correlation may occur because, in skull base surgeries, patients may experience postoperative hormonal or neurological alterations that result in symptoms not necessarily related solely to the specific nasal site.

Moreover, the period up to six months after surgery is marked by greater variations, with subsequent stabilization, as the condition of the nose. The SNOT-22, which assesses a broader range of symptoms, including domains not exclusively nasal, might capture these systemic alterations, whereas the ASK Nasal-12, being more focused on nasal symptoms, might not reflect these changes. This would explain the lack of statistical correlation between the two questionnaires at these specific times.

Currently, the SNOT-22 has been criticized for including domains that are not directly related to the specific nasal site, as well as for the time required for its completion. For this reason, new studies could consider the ASK Nasal-12 as a faster and more focused tool for assessing nasal function.

This way, this questionnaire will provide more and better studies on the quality of the nose-specific site and make surgeons worry about continually seeking to improve the surgical technique to improve the QOL offered to the patient.

## Conclusion

The translation, adaptation, and validation of the ASK Nasal-12 questionnaire into Brazilian Portuguese appear consistent and effective regarding its cultural equivalence for monitoring the QOL of patients undergoing transnasal access for endoscopic anterior skull base surgeries.

## Conflicts of interest

The authors declare no conflicts of interest.

## Acknowledgement

We would like to express our sincere gratitude to Andrew Little, the lead author and mentor of this new questionnaire, for his invaluable support and for endorsing this article. We also extend our thanks to Eduardo Macoto Kosugi, whose guidance in organizing the content and structuring this article was immensely helpful.
