# Supplementary figures and images for: Translation, adaptation, and validation of ASK nasal-12 into Brazilian Portuguese
Source: Braz J Otorhinolaryngol. 2024 Sep 14;91(1):101511. doi: 10.1016/j.bjorl.2024.101511 (PMC11492054; doi:10.1016/j.bjorl.2024.101511)

BJORL-D-24-00125 - Supplementary Material

Attachments 1.Anterior Skull Base Nasal Inventory.

**
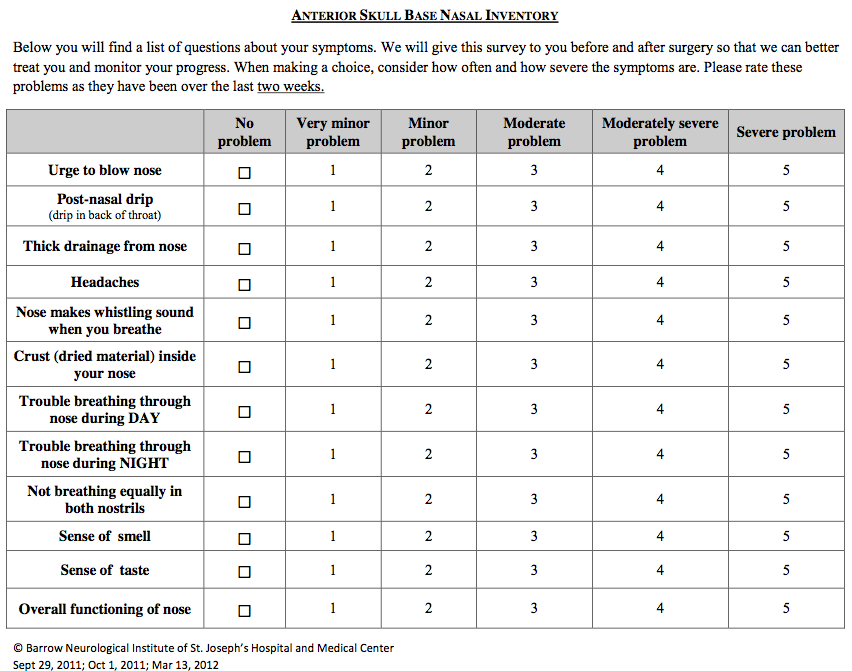
**

Supplement: Supplementary file 1 [file mmc1.doc]
